# Curcumin supplementation mitigates NASH development and progression in female Wistar rats

**DOI:** 10.14814/phy2.13789

**Published:** 2018-07-16

**Authors:** Rory P. Cunningham, Mary P. Moore, Angelique N. Moore, James C. Healy, Michael D. Roberts, R. Scott Rector, Jeffrey S. Martin

**Affiliations:** ^1^ Research Service‐Harry S Truman Memorial VA Hospital Columbia Missouri; ^2^ Department of Nutrition and Exercise Physiology University of Missouri Columbia Missouri; ^3^ Department of Biomedical Sciences Edward Via College of Osteopathic Medicine – Auburn Campus Auburn Alabama; ^4^ School of Kinesiology Auburn University Auburn Alabama; ^5^ Department of Medicine‐GI University of Missouri Columbia Missouri

**Keywords:** Curcumin, inflammation, NASH, steatosis

## Abstract

Curcumin, a naturally occurring plant polyphenolic compound, may have beneficial effects in nonalcoholic steatohepatitis (NASH) development. We examined whether curcumin supplementation could be used in both prevention and treatment of NASH with fibrosis. Female Wistar rats were provided ad libitum access to a “western diet” (WD) high in fat (43% kcal), sucrose (29% kcal), and cholesterol (2% w/v), as well as 15% fructose drinking water. Intraperitoneal CC1_4_ injections (0.5 mL/kg) were also administered at weeks 1, 2, 4, and 6 to accelerate development of a NASH with fibrosis phenotype. Rats were randomized to four groups (*n* = 9–12/group) and fed ad libitum: (1) WD for 8‐weeks (8WD), (2) WD enriched with curcumin for 8‐weeks (8WD+C; 0.2% curcumin, BCM‐95, DolCas Biotech) to assess prevention, (3) WD for 12‐weeks (12WD), (4) WD for 8‐weeks followed by 4‐weeks WD+C (12WD+C) to assess treatment. Curcumin prevention (8WD vs. 8WD+C) attenuated (*P* < 0.05) histological liver inflammation, molecular markers of fibrosis (Col1a1 mRNA) and a serum marker of liver injury (AST). Curcumin treatment (12WD vs. 12WD+C) reduced (*P* < 0.05) hepatocellular inflammation, steatosis, NAFLD Activity Scores, and serum markers of liver injury (AST, ALP). Moreover, curcumin treatment also increased hepatic pACC/ACC, ApoB100, and SOD1 protein, and decreased hepatic FGF‐21 levels; whereas, curcumin prevention increased hepatic glutathione levels. Both curcumin prevention and treatment reduced molecular markers of hepatic fibrosis (Col1a1 mRNA) and inflammation (TNF‐*α*, SPP1 mRNA). Curcumin supplementation beneficially altered the NASH phenotype in female Wistar rats, particularly the reversal of hepatocellular inflammation.

## Introduction

Nonalcoholic fatty liver disease (NAFLD) is a progressive disease of the liver that ranges on a wide pathological spectrum from simple hepatic steatosis to a more severe nonalcoholic steatohepatitis (NASH) phenotype, which can progress to fibrosis and cirrhosis (Buzzetti et al. 2016). Numerous factors are involved in the progression of NAFLD, including changes in lipid metabolism, insulin resistance, inflammatory cytokines, and oxidative stress (Brunt 2010). NAFLD has a high incidence rate, with reports in the general population ranging from 10% to 30% and as high as 80–100% in obese populations (Vernon et al. 2011; Chalasani et al. 2012). These rates exhibit gender‐specific differences, with males at a significantly higher risk of developing NAFLD relative to premenopausal women (Lonardo et al. 2006, 2015). Progression to NASH is the most rapidly increasing indication for liver transplantation in the United States (Wong et al. 2015), and is considered an independent risk factor for cardiovascular, liver‐related, and all‐cause mortality (Targher et al. 2008; Stepanova et al. 2013). Currently, there are no FDA‐approved pharmacological treatments for NASH.

Recently, dietary polyphenols have garnered much interest in their potential health benefits due to their wide range of possible therapeutic actions. Indeed, the main active component of turmeric (*Curcuma longa L*.) – curcumin, exhibits anti‐inflammatory, antioxidant, antifibrotic, and anticancer properties (Pari et al. 2008; Jurenka 2009). As increased hepatic inflammation and oxidative stress are characteristics of NASH development (Videla et al. 2004), curcumin supplementation may be a viable candidate as a potential therapeutic for this disease. Preliminary studies suggest curcumin may play a beneficial role in mitigating NASH development via anti‐inflammatory actions, as well as restoring balance to hepatic pro and antioxidant systems (Leclercq et al. 2004; Wu et al. 2008; Lee et al. 2017). Limited evidence also suggests that curcumin may have antifibrotic effects on the liver (Fu et al. 2008; Reyes‐Gordillo et al. 2008). Future studies are warranted to further examine this nutraceutical's beneficial effects on chronic liver disease.

Despite the sexual dimorphism of NAFLD, these is a paucity of research examining the effects of curcumin on NAFLD development in females. Moreover, while the majority of studies have centered around curcumin as a preventative supplement, we sought to examine whether curcumin can also rescue a NASH phenotype. Specifically, this study investigated whether supplementary dietary curcumin could be effective at both preventing the development and also treating NASH induced by a western diet (WD) and carbon tetrachloride (CCl_4_) administration in female Wistar rats.

## Methods

All experimental procedures were approved by Auburn University's Institutional Animal Care and Use Committee (IACUC, protocol # 2016‐2839, approval date 01 March 2016). Female Wistar rats at 3 months of age (~325 g) were purchased (Harlan Laboratories, Indianapolis, IN, USA) and allowed to acclimate in the animal housing facility at Auburn University for 5 months prior to experimentation. During acclimation, rats were pair housed and provided standard rodent chow (SC; 24% protein, 58% CHO, 18% fat; 114 Teklad Global #2018 Diet, Harlan Laboratories) and water ad libitum in a maintained ambient temperature and constant 12 h light: 12 h dark cycle. Following acclimation, at 8 months of age, the rats were randomly assigned to one of four groups (*n* = 12/group):

1. Rats were provided a WD that was custom modified for 2% cholesterol by weight (43% fat, 34% CHO, 23% protein, TD.160279; Envigo Teklad Diets, Madison, WI USA) for 8 weeks (8WD, prevention control group).
2. Rats were provided the same WD modified for 2% cholesterol by weight, but also supplemented with 0.2% Curcumin (WD+C) (BCM‐95; DolCas Biotech, LLC., Landing, NJ USA; TD.160280; Envigo Tekland Diets) for 8 weeks (8WD+C, prevention group).
3. Rats were provided the WD modified for 2% cholesterol by weight for 12 weeks (12WD, treatment control group).
4. Rats were provided the WD modified for 2% cholesterol by weight for 8 weeks followed by 4 weeks of the WD+C (12WD+C, treatment group).

Rats in all groups received intraperitoneal (IP) injections of CCl_4_ at 0.5 mL/kg of body mass at the start of weeks 1, 2, 4, and 6. Rats in all groups were provided with 15% fructose drinking water (15 g/100 mL) and the respective chow diet(s) for ad libitum consumption. During the study, body weight, food and fructose water consumption were measured weekly. The curcumin supplement (BCM‐95) containing 70% curcumin, 17% demethoxycurcumin, 3.5% bis‐demethoxycurcumin, and 7.5% turmeric essential oils, was provided by DolCas Biotech. Based on historical observations of daily food intake, a measure of 0.2% curcumin (i.e., BCM‐95) was selected in an attempt to obtain a dose of ~100 mg/kg of body weight per day. This dose has been used by others in order to elicit beneficial effects in rats treated with CCl_4_ injections (Park et al. 2000), and is also comparable to therapeutic dosages in human consumption. BCM‐95 ingestion for respective groups (8WD+C and 12WD+C) was also calculated at the conclusion of the study. For the duration of the study, all rats remained pair housed, unless cage mate deceased or required separation (due to fighting).

### Rats in 8 weeks experiment – prevention of NASH with curcumin experiment

For an 8‐week period following acclimation, rats were provided WD (8WD) or WD+C (8WD+C) ad libitum as described above (8WD *n* = 12, 8WD+C *n* = 12). One 8WD+C rat deceased (unknown reason), and was not included in the analyses. Thus, final group sizes were *n* = 12 and *n* = 11 for the 8WD and 8WD+C groups, respectively.

### Rats in 12 weeks experiment – treatment of NASH with curcumin Experiment

For a 12‐week period following acclimation, rats were provided WD ad libitum as described above (12WD *n* = 12). For an 8‐week period following acclimation, rats were provided WD followed by 4‐weeks of WD+C ad libitum as described above (12WD+C, *n* = 12). Two 12WD rats lost >20% body mass over the course of the study, and one 12WD+C rat developed a tumor and, thus, were euthanized for humane reasons and not included in the analyses. In addition, one 12WD+C rat deceased due to complications with CCl_4_ injection. Thus, final group sizes were *n* = 9 and *n* = 11 for the 12WD and 12WD+C groups, respectively.

### Necropsies and tissue preparation in rats from both feeding experiments

The night before necropsies, rats had their respective chow removed from their cage, but were provided tap water ad libitum in order to facilitate overnight fasting conditions. The following morning, rats were transported across campus to the Auburn University School of Kinesiology, and were allowed to acclimate to laboratory conditions for 2–3 h. Thereafter, rats were euthanized under CO_2_ gas in a 2 L induction chamber (VetEquip, Pleasanton, CA, USA). Following euthanasia, a final body mass was recorded, and blood was collected from the heart using a 22‐gauge syringe. Collected blood was placed in a 6‐mL serum separator tube and allowed to clot, centrifuged at 3500*g* for 10 min, and resultant serum was aliquoted into 1.7‐mL microcentrifuge tubes for storage at −80°C until analysis. Liver, heart, subcutaneous and omental fat, and gastrocnemius and triceps muscle tissues were dissected out, and tissue weights were recorded using a calibrated scale with a sensitivity of 0.0001 g (Mettler‐Toledo; Columbus, OH). The liver tissue was segmented into pieces for histology (~100 mg placed in a conical tube containing 5 mL of 10% formalin), RNA preservation (~30 mg placed in 500 *μ*L RNA/DNA Shield (Zymo Research, Irvine, CA, USA), and frozen at ‐20°C, and the remaining was flash‐frozen in liquid nitrogen and stored at −80°C for analytical procedures described below.

### Serum analyses

A serum aliquot was transferred to the Auburn University Veterinary Diagnostic Laboratory for analysis of serum total protein, albumin, globulin, alkaline phosphatase (ALP), aspartate aminotransferase (AST), and alanine aminotransferase (ALT) using an automated chemistry analyzer (Roche Cobas as C311; Roche Diagnostics, Indianapolis, IN).

### Liver homogenate assays

Liver homogenate assays were performed using total FGF21 (mouse/rat FGF21 kit Quantikine ELISA; Item No. MF2100, R&D Systems, Minneapolis, MN), superoxide dismutase activity (SOD; Item No. 706002, Cayman Chemical), and glutathione (Item No. 703002, Cayman Chemical) assay kits. Notably, liver tissue was homogenized as previously described (Rector et al. 2008), and assays were performed according to manufacturer's instructions.

### Western blotting

Liver homogenization and western blot analyses were conducted as previously described (Rector et al. 2008). The proteins evaluated were as follows: nuclear factor E2‐related factor 2 (NRF2; #137550, Abcam, Cambridge, MA), kelch‐like ECH‐associated protein 1 (KEAP1; #12721, Cell Signaling, Danvers, MA), acetyl‐CoA carboxylase (ACC; #3662, Cell Signaling) phosphorylated ACC (p‐ACC [Ser79]; #3661, Cell Signaling), fatty acid synthase (FAS; #3189, Cell Signaling), cluster of differentiation 36 (CD36; #133625, Abcam), microsomal triglyceride transfer protein (MTTP; #135994, Santa Cruz Biotechnology, Dallas, TX, USA), apolipoprotein B‐100 (ApoB100, #20797‐1, Abcam), fibroblast growth factor receptor substrate 2 (FRS2; #10425, Abcam), *β*‐Klotho (#74343, Santa Cruz Biotechnology), peroxisome proliferator‐activated receptor‐gamma (PGC1*α*; #3242, Millipore, St. Louis, MO), superoxide dismutase 1 (SOD1, #13498, Abcam), and superoxide dismutase 2 (SOD2, #13194, Cell Signaling). Blots (*n* = 9–12/group) were analyzed via densiometric analysis (Image Lab 3.0, Bio‐Rad, Hercules, CA, USA). Amido‐black staining was used to control for differences in protein loading and transfer as previously described (Rector et al. 2008).

### Liver mRNA analysis

Liver was removed from −80°C storage and crushed on a liquid nitrogen‐cooled stage. Approximately 30 mg of tissue was placed in 500 *μ*L of Ribozol (Ameresco), was homogenized by hand in microcentrifuge tubes using tight‐fitting pestles, and RNA isolation occurred according to manufacturer's instructions. Total RNA concentrations from isolated liver RNA were determined in duplicate using a NanoDrop Lite spectrophotometer (Thermo Fisher Scientific). One microgram of liver RNA was reversed transcribed into cDNA for RT‐PCR analysis with cDNA synthesis reagents (Quanta Biosciences, Gaithersburg, MD, USA) per the manufacturer's recommendations. Real time PCR was performed using gene‐specific primers and SYBR green chemistry (Quanta Biosciences). Primer sequences used were as follows: HDAC1 (housekeeping gene) forward primer (5′ → 3′): GAGCGGTGATGAGGATGAGG, reverse primer: CACAGGCAATGCGTTTGTCA; AP1 forward primer: CAGGTGGCACAGCTTAAACA, reverse primer: CGCAACCAGTCAAGTTCTCA; Colla1 forward primer: AGGCATAAAGGGTCATCGTG, reverse primer: ACCGTTGAGTCCATCTTTGC; COL1A2 forward primer: TTGACCCTAACCAAGGATGC, reverse primer: CACCCCTTCTGCGTTGTATT; SMA forward primer: ACCATCGGGAATGAACGCTT, reverse primer: CTGTCAGCAATGCCTGGGTA; FABP4 forward primer: TGAAATCACCCCAGATGACA, reverse primer: TCACGCCTTTCATGACACAT; JNK forward primer: CGGAACACCTTGTCCTGAAT, reverse primer: GAGTCAGCTGGGAAAAGCAC; NF‐*κ*B forward primer: TTGTCACTGCTGTCCCTCTG, reverse primer: GTGGGGACTGCGATACCTTA; NRF2 forward primer: CAGTCTTCACCACCCCTGAT, reverse primer: CCAAACTTGCTCCATGTCCT; SPP1 forward primer: GAGGAGAAGGCGCATTACAG, reverse primer: ATGGCTTTCATTGGAGTTGC; TNF‐*α* forward primer: GGTCAACCTGCCCAAGTACT, reverse primer: CTCCAAAGTAGACCTGCCCG; TGF‐*β* forward primer: TGAGTGGCTGTCTTTTGACG, reverse primer: TGGGACTGATCCCATTGATT. Fold change values relative to 8WD rats were determined using 2^−ΔΔCT^ method where: (1) 2^−ΔCT^ = HDAC1 CT − gene of interest CT, and (2) 2^ΔΔCT^ (or fold‐change) = [−2^−ΔCT^ value of 8WDT/12WD/8WD4T rat/2^−ΔCT^ average of 8WD group]. Of note, the housekeeping gene (i.e., HDAC1) remained stable across all treatments, and melt curve analyses were performed during each PCR reaction to confirm that only one PCR product was obtained.

### Liver pathology

Formalin‐fixed liver specimens were transferred to Veterinary Diagnostics Pathology, LLC (Fort Valley, VA, USA) for analysis. To examine liver morphology, formalin‐fixed, paraffin‐embedded livers were sectioned and stained with hematoxylin and eosin (H&E). To examine collagen deposition/fibrosis, additional slides were stained with trichrome. Liver specimens were graded and scored by an independent pathologist for changes in fat accumulation, ballooning, inflammation, and fibrosis.

### Statistical analysis

Statistical analyses were completed in SPSS (IBM SPSS Statistics for Windows, Version 24.0. Armonk, NY) with *P* < 0.05 used to determine statistical significance of all comparisons. Comparisons were made between 8WD versus 8WD+C and 12WD versus 12WD+C to focus on prevention and treatment comparisons. Homogeneity of variance was assessed by Levene's Test for Equality of Variances. If significant, the pooled standard deviation was not used to determine group differences, but instead the Welch–Satterthwaite method was used, where equal variances are not assumed. In cases where there was homogeneity of variances, a Student's independent *t*‐test was run on the data to determine significance. Data are presented as means ± SE (*n* = 9–12 observations are represented per group).

## Results

### Animal characteristics

Animal characteristics are presented in Table 1. Final body mass (g), food intake (g/week), and liver mass (g) were not significantly different for 8WD versus 8WD+C or 12WD versus 12WD+C (*P* > 0.05). Daily average BCM‐95 intake was 21.4 ± 3.5 mg/day for weeks in which respective rats consumed the diet supplemented with BCM‐95 (20.4 ± 4.0 mg/day and 22.2 mg/day for 8WD+C and 12WD+C, respectively). Relative to body mass, daily average BCM‐95 intake was 71.3 ± 19.8 mg/kg/day for weeks in which respective rats consumed the diet supplemented with BCM‐95 (67.9 ± 27.3 mg/kg/day and 75.3 ± 9.5 mg/kg/day for groups 8WD+C and 12WD+C, respectively).

**Table 1:** Animal characteristics are presented in table 1 Initial body mass (g), final body mass (g), food intake (g/week), and liver mass (g) were not significantly different for 8WD versus 8WD+C and 12WD versus 12WD+C (*P* > 0.05)

Variable	8WD	8WD+C	12WD	12WD+C
Initial body mass (g)	382.33 ± 8.60	418.45 ± 17.71	390.67 ± 18.18	373.09 ± 18.99
Final body mass (g)	389.35 ± 9.78	429.78 ± 21.18	459.78 ± 21.38	441.57 ± 21.50
Food intake (g/week)	219.79 ± 8.31	199.05 ± 5.52	277.88 ± 19.61	249.55 ± 12.27
Liver mass(g)	15.66 ± 1.00	17.87 ± 1.54	17.44 ± 1.368	15.41 ± 1.33

Values are mean ± SE (*n* = 9–12 per group), g = grams.

### Liver phenotype

**Figure 1:**
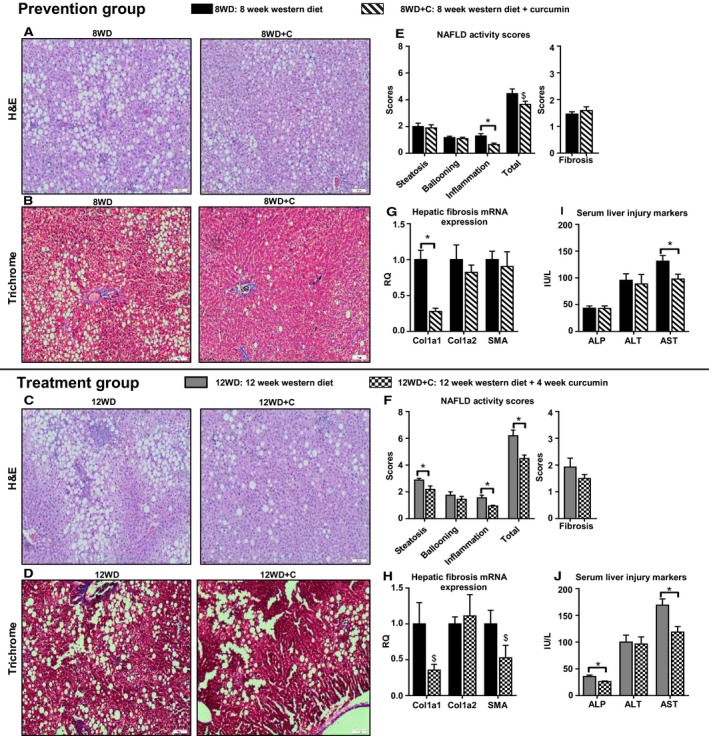
Effect of curcumin on liver phenotype: histological steatosis and inflammation (A) 8WD versus 8WD+C and (C) 12WD versus 12WD+C, histological fibrosis (B) 8WD versus 8WD+C and (D) 12WD versus 12WD+C, Nonalcoholic fatty liver disease (NAFLD) activity scores and fibrosis scores (E) 8WD versus 8WD+C and (F) 12WD versus 12WD+C, Hepatic Fibrosis Markers (G) 8WD versus 8WD+C and (H) 12WD versus 12WD+C, serum liver injury markers (I) 8WD versus 8WD+C and (J) 12WD versus 12WD+C. Values are mean ± standard error (*n* = 9–12). * denotes significant difference *P* < 0.05 between 8WD versus 8WD+C and 12WD versus 12WD+C. $ denotes trend toward significance *P* < 0.100.

Overall, Total NAFLD Activity Scores (NAS) trended toward significance (*P* = 0.079) for the prevention group (8WD vs. 8WD+C) and was significantly lower for the treatment group (12WD vs. 12WD+C) (*P* < 0.05, Fig. 1E–F), with lower scores observed for the groups who received curcumin. Curcumin reduced hepatocellular inflammation for both 8WD+C and 12WD+C versus 8WD and 12WD, respectively (*P* < 0.05, Fig. 1E–F). Additionally, hepatic steatosis was significantly reduced in the curcumin treatment group (12WD vs. 12WD+C) (*P* < 0.05, Fig. 1). There was no significant difference between fibrosis for either groups (*P* > 0.05, Fig. 1E–F). Col1a1 mRNA expression, a marker of collagen deposition, was significantly lowered in the prevention group (*P* < 0.05, Fig. 1G), whereas Col1a1 and SMA trended toward significance in the treatment group (*P* = 0.068 and *P* = 0.089, respectively, Fig. 1H). There were no changes in Col1a2, another measure of collagen deposition for either groups (*P* > 0.05, Fig. 1G–H). Regarding liver injury markers, curcumin lowered serum AST versus control (8WD vs. 8WD+C and 12WD vs. 12WD+C, *P* < 0.05). Additionally, ALP was significantly lower for the treatment group (12WD vs. 12WD+C) (*P* < 0.05, Fig. 1I–J).

**Figure 2:**
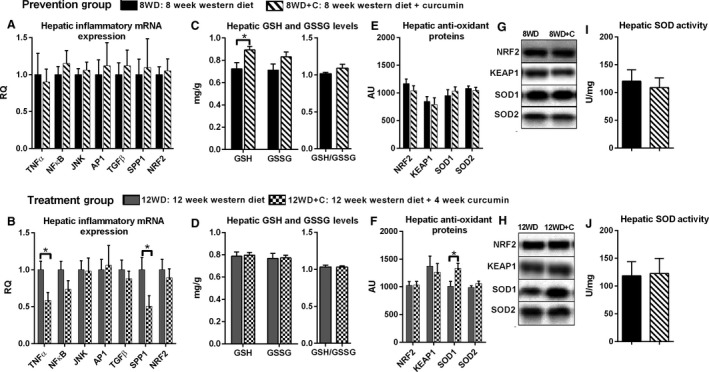
Effect of curcumin on inflammation, antioxidant, and SOD activity: hepatic inflammatory mRNA expression (A) 8WD versus 8WD+C and (B) 12WD versus 12WD+C, hepatic glutathione and oxidized glutathione levels, and glutathione/oxidized glutathione ratio (C) 8WD versus 8WD+C and (D) 12WD versus 12WD+C, hepatic antioxidant proteins (E) 8WD versus 8WD+C and (F) 12WD versus 12WD+C, representative Western Blots (G) and (H), hepatic SOD activity (I) 8WD versus 8WD+C and (J) 12W versus 12WD+C. Values are mean v standard error (*n* = 9–12). * denotes significant difference *P* < 0.05 between 8WD versus 8WD+C and 12WD versus 12WD+C.

Increased hepatic inflammation and oxidative stress are hallmarks of NASH progression. Accordingly, we sought to determine the effect of curcumin on markers of hepatic and systemic inflammation. Treatment with curcumin (12WD vs. 12WD+C) significantly reduced the mRNA expression of hepatic inflammatory cytokines, including TNF*α* and SPP1 (*P* < 0.05, Fig. 2B). There were no significant changes in NF‐*κ*B, JNK, AP1, TGF*β* or NRF2 mRNA levels for 12WD versus 12WD+C (*P* > 0.05, Fig. 2B).

Curcumin may have an antioxidant effect in the setting of NAFLD/NASH, hence we assessed common markers of oxidative stress. Supplementation with curcumin significantly increased glutathione (GSH) levels in the prevention group (8WD vs. 8WD+C) (*P* < 0.05, Fig. 2C), and tended to increase GSH to oxidized glutathione (GSSG) ratio (GSH/GSSG) in the same group (*P* = 0.117, Fig. 2C). Treatment with curcumin resulted in no changes in GSH or GSSG levels, and GSH/GSSG ratio (12WD vs. 12WD+C) (*P* > 0.05, Fig. 2D). Proteins involved in antioxidant defense including NRF2 and KEAP1 were not altered with curcumin supplementation (*P* > 0.05, Fig. 2E–F). Curcumin also had no significant effect on SOD activity, or hepatic SOD1 or SOD2 expression for the prevention group (8WD vs. 8WD+C) (*P* > 0.05, Fig. 2E and I). Similarly, there were no significant changes in SOD activity or SOD2 content in the liver for the treatment group (12WD vs. 12WD+C) (*P* > 0.05, Fig. 2F and J). However, curcumin treatment (12WD vs. 12WD+C) significantly increased hepatic SOD1 protein content (*P* < 0.05, Fig. 2F).

**Figure 3:**
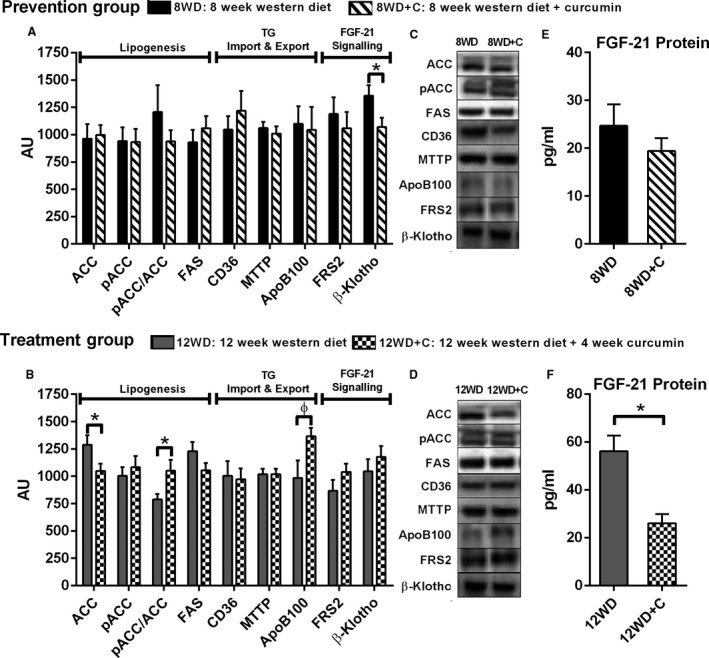
Effect of curcumin on lipogenesis, triglyceride (TG) import and export and FGF‐21: lipogenesis (ACC, pACC, pACC/ACC ratio, FAS), TG import and export (CD36, MTTP, APOB100) and FGF‐21 signaling (FRS2, *β*‐klotho) (A) 8WD versus 8WD+C and (B) 12WD versus 12WD+C, representative Western blots (C) and (D), liver FGF‐21 protein (E) 8WD versus 8WD+C and (F) 12WD versus 12WD+C. Values are mean ± SE (*n* = 9–12). * denotes significant difference *P* < 0.05 between 8WD versus 8WD+C and 12WD versus 12WD+C. *ɸ* denotes trending toward significance *P* = 0.064.

It is understood that hepatic lipogenesis and lipid regulation can change in the presence of disease, hence mechanisms involved in hepatic lipid regulation were analyzed to determine how they were influenced by curcumin supplementation. Treatment with curcumin (12WD vs. 12WD+C) significantly lowered total ACC, a marker of hepatic lipogenesis, and ApoB100, a marker of triglyderide (TG) export (*P* < 0.05, Fig. 3B). However, curcumin had no effect on protein markers of lipogenesis, TG import and TG export for the prevention group (8WD vs. 8WD+C) (*P* > 0.05, Fig. 3A).

Fibroblast growth factor 21 (FGF‐21) is a hormone associated with energy homeostasis, but has recently emerged as a plasma marker for NAFLD development (Dushay et al. 2010; Li et al., 2010). In the prevention group, curcumin was associated with significantly lower levels of the FGF‐21 signaling protein *β*‐klotho in the liver compared to control rats (8WD vs. 8WD+C) (*P* < 0.05, Fig. 3B). However, no changes in hepatic FGF‐21 protein were observed for this group (*P* > 0.05, Fig. 3E). Finally, curcumin treatment significantly lowered hepatic FGF‐21 protein (*P* < 0.05, Fig. 3F) in the absence of changes in FRS2 or B‐klotho (*P* > 0.05, Fig. 3B) compared to control rats (12WD vs. 12WD+C).

## Discussion

Polyphenols such as curcumin may serve as a potential therapeutic to mitigate the development of NASH, as they have been shown to possess anti‐inflammatory and antioxidant properties. In this study, we demonstrate that supplemental dietary curcumin attenuated the development of NAFLD progression in female Wistar rats via a reduction in hepatic steatosis and inflammation. Importantly, these data further explore the beneficial effects of curcumin on both the prevention and treatment of chronic liver disease, while providing novel evidence for its effects on NASH progression in female rats.

Rats fed a WD for 8 or 12 weeks displayed pronounced hepatic steatosis, hepatocyte ballooning, and hepatocellular inflammation, resulting in elevated NAS. Curcumin prevention, referring to 8WD versus 8WD+C, lowered inflammation and NAS, whereas curcumin treatment (12WD vs. 12WD+C) reduced steatosis and inflammation, significantly reducing NAS compared to 12WD. Curcumin has been shown to reduce steatosis in the liver (Lee et al. 2017), although this was accompanied by significant attenuation of weight gain. Here, we demonstrate that curcumin reduced hepatic steatosis in the absence of weight loss. Additionally, dietary curcumin attenuated the increases in AST in the prevention and treatment groups, and ALP in the treatment group. This ability of curcumin to reduce serum markers of liver injury seen in this study is similar to previous reports (Kaur et al. 2006; Ramirez‐Tortosa et al. 2009). However, the lack of changes observed in serum ALT levels in both groups is not entirely clear, but likely relates to the dosage of curcumin supplementation given compared with previous reports (Park et al. 2000). Regardless, this study demonstrates significant reduction in hepatocellular inflammatory cell infiltration and reduced NAFLD Activity Score with curcumin supplementation.

NF‐*κ*B is a transcription factor that plays a vital regulatory role in the inflammatory and immune pathways, and its activation results in the progression of steatosis to NASH (Malaguarnera et al. 2009). A common pathway by which curcumin has been shown to exert its beneficial effects is through mitigating the hepatic NF‐*κ*B pathway (Leclercq et al. 2004; Weisberg et al. 2008; Li et al. 2010). Surprisingly, curcumin had no effect on markers of NF‐*κ*B signaling in this study with either prevention or treatment groups, despite reduced histological inflammation in both and lower steatosis scores in the treatment group. This is in contrast to previous work where curcumin reduced NF‐kB and inflammatory signals in a methionine and coline deficient (MCD) diet model of NASH (Leclercq et al. 2004).

Curcumin significantly reduced a number of hepatic gene and serum inflammatory markers in both the treatment and prevention groups. SPP1 codes for the protein osteopontin and its upregulation has been implicated in the progression to NASH via its positive feedback loop on inflammatory cytokines (Sahai et al. 2004). Treatment with curcumin significantly lowered hepatic SPP1 mRNA expression possibly further attenuating inflammation. While no differences in fibrosis were observed in either group, key fibrotic inflammatory signals such as TNF*α* and Col1a1 mRNA were lowered with curcumin supplementation. These well‐established profibrotic markers have been implicated in the progression of NASH and fibrosis development (Palacios et al. 2008; Braunersreuther et al. 2012). Curcumin may be a novel method of modulating these gene markers in attenuating NASH development.

Oxidative stress as a result of increased reactive oxygen species (ROS) production is well‐known to play a role in the pathogenesis of NASH. Hepatocytes are continually exposed to and protected from oxidative stress and injury by a range of antioxidant pathways. Imbalances between these prooxidant and antioxidant processes can result in increased oxidative stress (Videla et al. 2004). Glutathione (GSH) is one such antioxidant that is known to be depleted in NAFLD (Lieber et al. 2004). Here, we report that curcumin prevention increased free hepatic GSH levels compared to the 8WD group, suggesting an increased scavenging ability of free radicals with curcumin prevention. Additionally, nuclear factor‐erythroid‐2‐related factor 2 (NRF2) plays a key role in the antioxidant defense system as a transcription factor for many antioxidant enzymes (Li et al. 2008). Surprisingly, neither NRF2 nor its repressor, Kelch‐like ECH‐associated protein 1 (KEAP1), were affected by curcumin supplementation. However, superoxide dismutase 1 (SOD1), a downstream target of NRF2 and important mediator of antioxidant defense, was significantly increased with curcumin treatment. SOD activity has been shown to be decreased in humans diagnosed with NASH, suggesting an impairment of the antioxidant enzymatic defense system in NASH (Koruk et al. 2004). In addition, overexpression of SOD1 in the liver of db/db mice significantly reduced hepatic ROS (Kumashiro et al. 2008), whereas curcumin aided in the reactivation of hepatic SOD in male rats with CCl_4_ induced liver injury (Wu et al. 2008). The findings of this study confirm the antioxidant benefits of curcumin supplementation.

The initial development of hepatic steatosis can be attributed to, in part, by alterations in the molecular mediators that regulate hepatic lipid accumulation. Increased de novo lipogenesis, impaired fatty acid *β*‐oxidation, increased lipid import and decreased export all contribute to lipid accumulation in the liver (Browning and Horton 2004). Indeed, obese patients with NAFLD exhibit decreased very‐low‐density‐lipoproteins (VLDL)‐ApoB100 secretion rates (Charlton et al. 2002), contributing to hepatic TG accumulation, whereas CCl_4_ administration causes depressed secretion of VLDL (Boll et al. 2001). Curcumin treatment significantly increased the protein markers of pACC/ACC and ApoB100, suggesting decreased lipid import and increased lipid export in the liver. ACC is one of the key enzymes involved in de novo lipogenesis and hepatic lipid storage (Kim et al. 2017), and its phosphorylation causes inactivation of the enzyme. Taken together, these findings help explain the reduction in hepatic steatosis observed with curcumin treatment.

Another potential mechanism by which curcumin exerted its benefits is through the modulation of fibroblast growth factor 21 (FGF‐21). This hepatokine is produced and released from the liver upon fasting in an effort to control blood glucose levels via facilitation of fatty acid oxidation and gluconeogenesis (Liang et al. 2014). Paradoxically, elevated plasma FGF‐21 levels are commonly seen in obese humans and animals (Zhang et al. 2008; Chui et al. 2010), indicating a FGF‐21 resistant state. Additionally, emerging evidence suggests that FGF‐21 may be a biomarker for NAFLD, as elevated serum levels are seen in patients with NAFLD as well as correlated with hepatic TG (Dushay et al., 2010; Li et al. 2010). Here, we show that 12 weeks of WD caused significant elevations in FGF‐21 levels, indicative of FGF‐21 resistance, whereas curcumin treatment attenuated this increase. This suggests that curcumin may help reverse FGF‐21 resistance, a finding supported by others (Zeng et al. 2016).

A common problem with testing the efficacy of human health supplements on animals is comparing efficacious and species‐specific dosages. Nontoxic consumption of curcumin in humans has been as high as 12 g per day and reportedly well tolerated with sufficient absorption (Vareed et al. 2008; Preitner et al. 2009). Here, rats consumed roughly 71.3 mg/kg of curcumin per day, which equates to just under 5 g per day for a 70 kg person, well within the safe and tolerable range. This highlights the safety and practicality of this supplement as a means for potential treatment for NAFLD, of which there is no current pharmacological treatment. Vitamin E supplementation has shown some benefit in the treatment of NASH in clinical trials (Sanyal et al. 2010), although no such trial has been completed to examine the effect of curcumin on this population. Notably, curcumin is reported to have at least 10 times the antioxidant power of vitamin E (Kowluru and Kanwar 2007), further highlighting the need for future trials of this supplement on NAFLD development.

In summary, we report that curcumin supplementation can be effective in partially mitigating the development of NASH and perhaps more effective in treating NASH via a reduction in hepatic steatosis and hepatocellular inflammation in female rats. With no FDA‐approved pharmacological therapies for NAFLD currently available, curcumin supplementation may provide an economic, safe, and practical remedy for the attenuation of NASH progression. Future clinical trials should seek to examine the effectiveness of curcumin in chronic liver disease.

## Conflicts of Interest

The authors have no conflicts of interest to disclose.
